# Perovskite photovoltaics: stability and scalability

**DOI:** 10.1038/s41598-023-31512-z

**Published:** 2023-03-16

**Authors:** Shuxia Tao, Lakshminarayana Polavarapu, Paola Vivo

**Affiliations:** 1 Materials Simulation and Modelling, Department of Applied Physics, Eindhoven University of Technology, 5600 MB Eindhoven, The Netherlands; 2 Materials Chemistry and Physics, Department of Physical Chemistry, CINBIO, Universidade de Vigo, Campus Universitario Lagoas Marcosende, 36310 Vigo, Spain; 3 Hybrid Solar Cells, Faculty of Engineering and Natural Sciences, Tampere University, P.O. Box 541, 33014 Tampere, Finland

**Keywords:** Solar cells, Electronic properties and materials

## Abstract

Perovskite solar cells must overcome the long-term stability problem in order to be put into practical use. Materials science, through the development of synthetic chemistry, materials characterization and device engineering can contribute to improvements in stability and scalability towards enabling large scale production. This Collection presents recent research efforts in stabilizing perovskite solar cells with three interconnected themes: characterizing instability, synthesizing stable perovskites and curing the interfaces.

Currently, one of the most fascinating areas of energy materials and optoelectronic devices research is perovskite solar cells. They are not only found to be one of the most efficient types of solar cells, but also constitute one of the fastest growing fields in materials science. Yet, with all the momentum in the field, perovskite solar cells are not ready to be commercialized, mainly due to the long-term instability problems^1–6^. These problems have their atomistic origin; the materials are mixed covalent and ionic and they have soft and dynamic lattices. Such characteristics have direct consequences for the long-term stability of the solar cells, including relatively fast degradation of perovskite absorbers^1,7–10^, their interfaces with charge transport layers under thermal stress^2,5,6^, photoexcitation and electrical bias, and the hysteresis of current–voltage (J–V) curves^3,4^. This Collection includes recent research efforts in stabilizing perovskite solar cells with three interconnected themes: (1) characterization methods for studying the instability problems from atomistic to device level^1–4^, (2) strategies to improve the stability and cure the interfaces problems at the perovskite/charge transport layers^5,6^ in the devices (3) materials engineering strategies to synthesize and fabricate stable perovskites in the lab^7,8,10^ and industrial scale^9^.

## Characterizing instability

To stabilize perovskite solar cells, fundamental research focusing on understanding the origin of instability through different characterizations is essential. Several articles focus on the ion migration-induced effect on perovskite absorbers, on the aging at electrode/charge transport layer/perovskite interfaces, and hysteresis behavior in the devices. For instance, Cuzzupè et al.^1^ employed a physical contact annealing approach to probe the A-site cation diffusion between MAPbI_3_ and FAPbI_3_ films and find a mutual cation migration. The authors highlight that the migration is helpful to convert the unwanted yellow phase of FAPbI_3_ to the black phase and heals MAPbI_3_ by removing pinholes. Yet, similar phenomena can be detrimental to device application when other stress factors, such as hysteresis in J–V under the operation of the solar cells^3–6^ are present. In this regard, Minbashi et al.^3^ studied such an effect by using a drift–diffusion solver with an ion migration model incorporated. They found that the scan rate and charge-carrier mobility have a significant effect on the severity of the hysteresis. Furthermore, Huang et al.^4^ argue that the dielectric constant of the charge transport layers and the total ion density, relevant to the quality of the perovskite crystal and the degree of degradation of the interfaces, are also important factors. These factors contribute to the strength of the built-in electric field across the perovskite and its interfaces with contact layers, which in turn affect the degradation speed of the perovskites and interfaces.

## Growing stable perovskites

Obtaining high-quality perovskite films with fewer defects has recently attracted extensive attention as a way to improve the stability of corresponding solar cells. Four articles in the Collection focus on this theme^7–10^. Two articles are dedicated to the use of additives to control crystallization and nucleation^7,8^. Where Eze et al*.*^7^ employ AgI as a nucleation promotor in the fabrication of CsPbIBr_2_ perovskites with increased grain size and reduced defects, Koech et al.^8^ improve the film morphology and the interfacial energy alignment by incorporating a small amount of polyethylene oxide in the hybrid FAPbI_3_ perovskite. In addition, efforts have also been devoted to the optimization of fabrication methods to suit industrial-scale production. For instance, Rezaee et al.^9^ developed perovskite precursor inks that are compatible with a scalable slot-die coating method at room temperature. They demonstrated the importance of the solvents, annealing temperature, air or inert gas blowing on the nucleation and growth mechanisms. On the other hand, a sequential spray deposition technique has been used to fabricate stacked triple cation perovskites with graded Cs content, enabling a wide absorption range and broadband photodetector applications^10^. Although this work does not focus directly on solar cells, it demonstrates that the careful control of the deposition process of perovskites opens up novel applications in other optoelectronic devices.

## Curing the interfaces

The degradation of the interface of the perovskite-charge transport layer is another very challenging issue for the stability. In particular, the high efficiency cells often employ organic materials, Spiro-MeOTAD and PEDOT:PSS as charge transport layers^5,6^, which degrade severely and cause the J–V hysteresis and thus leads to rapid decrease of the efficiency of the solar cells. Two articles in this Collection propose to either replace the organic hole transport layer with an inorganic one (ZnCo_2_O_4_ nanoparticles), by Jheng et al.^5^ or to use an inert interlayer (polymer polyaniline) in between perovskite and electron transport layer, by Kim et al.^6^. Both approaches resulted in improved stability. It should be noted that the addition of a new layer or replacing an existing layer introduces more complexity to the device and may come at a cost of other desired properties. Further fundamental understanding of the functions and the impact of these new layers and adjustment of other adjacent layers may be required.

The field of perovskite solar cells has revolutionized the paths for development of energy materials. The simultaneous push for high-efficiency and quest into fundamental materials chemistry and physics results in an exciting and fast-developing field. It is our opinion that commercial perovskite solar cells can be seen in the near future only if improved performance can be translated to large area devices with greater stability, and this requires sound knowledge at the fundamental level of materials science. This research not only provides insights on which knobs to turn in the design of stable and efficient solar cells but also demonstrates the power of materials science to develop renewable energy technologies.

We thank all the authors for their contributions and hope that researchers working on perovskite solar cells and other photovoltaic technologies will enjoy reading this Collection. We also thank the editorial staff of *Scientific Reports* for their kind support.
